# Clozapine once- versus multiple-daily dosing: a two-center cross-sectional study, systematic review and meta-analysis

**DOI:** 10.1007/s00406-022-01542-1

**Published:** 2022-12-29

**Authors:** Nazar Kuzo, Ekkehard Haen, Dominic M. Ho, Hiroyoshi Takeuchi, Marianna Piras, Chin-Bin Eap, Renato de Filippis, Philipp Homan, John M. Kane, Marc-André Roy, Michael Paulzen, Georgios Schoretsanitis

**Affiliations:** 1 Department of Psychiatry, Psychotherapy and Psychosomatics, Psychiatry University Hospital Zurich, University of Zurich, Zurich, Switzerland; 2 Department of Cardiology, University Heart Center, University Hospital Zürich, Zurich, Switzerland; 3 Clinical Pharmacology, Department of Psychiatry and Psychotherapy and Department of Pharmacology and Toxicology, University of Regensburg, Regensburg, Germany; 4 Department of Neuropsychiatry, Keio University School of Medicine, Tokyo, Japan; 5 Unit of Pharmacogenetics and Clinical Psychopharmacology, Center for Psychiatric Neuroscience, Department of Psychiatry, Lausanne University Hospital, University of Lausanne, Lausanne, Switzerland; 6 School of Pharmaceutical Sciences, University of Geneva, Geneva, Switzerland; 7 Psychiatry Unit, Department of Health Sciences, University Magna Graecia of Catanzaro, Catanzaro, Italy; 8 Department of Psychiatry, The Zucker Hillside Hospital, Northwell Health, Glen Oaks, NY USA; 9 Center for Psychiatric Neuroscience, The Feinstein Institute for Medical Research, Manhasset, NY USA; 10 Département de Psychiatrie et Neurosciences, Université Laval, Québec City, QC Canada; 11 Department of Psychiatry, Psychotherapy and Psychosomatics, JARA Translational Brain Medicine, RWTH Aachen University, Aachen, Germany; 12 Center for Research and Innovation in Clinical Pharmaceutical Sciences, Lausanne University Hospital, University of Lausanne, Lausanne, Switzerland; 13 Institute of Pharmaceutical Sciences of Western Switzerland, University of Geneva, University of Lausanne, Geneva, Switzerland; 14 Department of Psychiatry and Molecular Medicine, Zucker School of Medicine at Hofstra/Northwell, Hempstead, NY USA; 15 Centre de Recherche CERVO, Quebec City, QC Canada; 16 Alexianer Hospital Aachen, Aachen, Germany

**Keywords:** Treatment-resistant schizophrenia, Clozapine, Divided dosing, Antipsychotics, Polypharmacy

## Abstract

Evidence regarding effectiveness and safety of clozapine once- vs. multiple-daily dosing is limited. We compared demographic and clinical parameters between patients with once- vs. multiple-daily dosing in the Department of Psychiatry and Psychotherapy, University of Regensburg, Germany (AGATE dataset), and the Department of Psychiatry, Lausanne University Hospital, Switzerland, using non-parametric tests. Effectiveness and safety outcomes were available in the AGATE dataset. We performed a systematic review in PubMed/Embase until February 2022, meta-analyzing studies comparing clozapine once- vs. multiple-daily-dosing. We estimated a pooled odds ratio for adverse drug-induced reactions (ADRs) and meta-analyzed differences regarding clinical symptom severity, age, percentage males, smokers, clozapine dose, and co-medications between patients receiving once- vs. multiple-daily dosing. Study quality was assessed using the Newcastle–Ottawa-Scale. Of 1494 and 174 patients included in AGATE and Lausanne datasets, clozapine was prescribed multiple-daily in 74.8% and 67.8%, respectively. In the AGATE cohort, no differences were reported for the clinical symptoms severity or ADR rate (*p* > 0.05). Meta-analyzing eight cohorts with a total of 2810 clozapine-treated individuals, we found more severe clinical symptoms (*p* = 0.036), increased ADR risk (*p* = 0.01), higher clozapine doses (*p* < 0.001), more frequent co-medication with other antipsychotics (*p* < 0.001), benzodiazepines (*p* < 0.001), anticholinergics (*p* = 0.039), and laxatives (*p* < 0.001) in patients on multiple- vs. once-daily dosing. Of six studies, five were rated as good, and one as poor quality. Patients responding less well to clozapine may be prescribed higher doses multiple-daily, also treated with polypharmacy, potentially underlying worse safety outcomes. Patient preferences and adherence should be considered during regimen selection.

**Supplementary Information:**

The online version contains supplementary material available at 10.1007/s00406-022-01542-1.

## Introduction

Prescribing antipsychotics once-daily may be convenient for patients and eagerly adopted by clinicians [1–3]. Specifically, a recent meta-analysis comparing once- vs. multiple-daily dosing regimens for various psychotropic agents reported a better safety profile while preserving comparable efficacy in patients prescribed antipsychotics once-daily [4]. The pharmacological aspects of the debate between once- vs multiple-daily dosing regimens include the half-life of the prescribed medications [5, 6], for example, for antipsychotics with short half-lives, i.e., less than 24 h, efficacy may benefit from dividing dosing over day [7], although morning intake of a sedating antipsychotic may cause daytime sleepiness, which may impair functioning.

This may also hold for clozapine, which is the only antipsychotic agent approved for the indication of treatment-resistant schizophrenia (TRS) [8, 9] and has a half-life of 12 h at steady state [10]. For example, splitting of the maintenance clozapine dose of 150–300 mg/day is being recommended in Canada with an option of a single-dose administration if the daily dose is lower than 200 mg/day [10], while dividing daily clozapine is recommended in the US for target daily doses ranging above 300–450 mg/day [11]. In Japan, divided dosing is recommended already above 50 mg/day [12]. These differences regarding recommendations for clozapine dose splitting may result in different prescription patterns worldwide with different percentages of patients being treated with clozapine once vs. multiple times per day in different countries [13, 14].

Furthermore, the available evidence regarding the effectiveness and safety outcomes for different clozapine dosing regimens currently derives from two cross-sectional studies [13, 14]. First, Takeuchi et al. compared groups of patients treated with clozapine once- vs. multiple-daily dosing in two large psychiatric hospitals in North America [13]; researchers suggested that clozapine was prescribed once-daily at doses even higher then 200–300 mg/d without differences for the effectiveness or reported clozapine-related seizures between patients with once- vs. multiple-daily dosing [13]. In a more recent cross-sectional study in two large Japanese psychiatric hospitals, Kitagawa et al. did not find any differences for clinical response or tolerability outcomes between patients with once- vs. multiple-daily dosing [14]; however, prevalence of depression/anxiety symptoms was lower in patients with divided vs. once-daily, which may have been mainly attributed to placebo effects [14]. Moreover, this study did not report any differences for peak and trough clozapine plasma levels between patients with divided vs. once-daily dosing, contrasting the assumptions of a previous pharmacokinetic simulation study [6]. This early pharmacokinetic simulation study had suggested that because of differences regarding pharmacokinetic variation, multiple-daily dosing might be associated with better effectiveness and tolerability outcomes compared to once-daily dosing of clozapine [6].

The aim of our study was to cross-sectionally compare the effectiveness and safety of clozapine prescribed once- vs. multiple-daily dosing in two different European cohorts. Further, we systematically reviewed and meta-analyzed available data on effectiveness and safety outcomes from previous comparisons between different clozapine dosing regimens.

## Materials and methods

### Study design and setting

Data collection was performed in two centers: the Department of Psychiatry and Psychotherapy at the University of Regensburg, Germany, and the Department of Psychiatry of the Lausanne University Hospital, Switzerland. In the Regensburg center, data was collected between 2005 and 2015 as part of the standard clinical practice of the AGATE (Arbeitsgemeinschaft Arzneimitteltherapie bei psychischen Erkrankungen), a non-profit working group aiming to improve drug efficacy and safety in the treatment of mental illnesses [15]. Therefore, we refer to it as AGATE cohort. For both available datasets, reporting adheres to the Strengthening the Reporting of Observational Studies in Epidemiology (STROBE) guidelines (Supplementary table S1) [16].

#### AGATE (Arbeitsgemeinschaft Arzneimitteltherapie bei psychiatrischen Erkrankungen) cohort

A dataset containing clinical outcomes, demographic characteristics, therapeutic regimen information and plasma concentrations of clozapine of 1644 in- and outpatients with a broad spectrum of psychiatric diseases was used. Treating clinicians assessed clinical response using the Clinical Global Impressions (CGI) scale [17, 18] as a part of usual case. The CGI scale consists of two one-item measures: one for the severity (CGI-S), which was assessed by treating clinicians at the admission timepoint, and one for improvement (CGI-I), which was assessed by treating clinicians at the discharge timepoint. In the group of responders, we included patients with CGI-I values of 1 (very much improved) or 2 (much improved), whereas non-responders were patients with a CGI-I > 2. Raters were blinded for plasma levels of clozapine, as clinical ratings were performed at the same time when blood samples were collected. Assessment of safety was based on detailed narrative reports of adverse drug-induced reactions (ADRs) provided by treating clinicians. Additionally, information about ADRs was classified according to domains of Udvalg for kliniske undersogelser-Scale (UKU) [19]. Blood samples were drawn just before clozapine administration reflecting trough concentrations at steady-state conditions. A validated high-performance liquid chromatography with ultraviolet detection was used [20]. The laboratory regularly runs internal quality controls and participates in external quality assessment schemes by INSTAND (Düsseldorf, Germany, www.instandev.de). Data registration followed standardized protocols [21]. The following data were extracted: age, sex, body mass index (BMI), smoker status, clozapine dosing regime, clozapine daily dose, as well as concomitant use of other antipsychotics, benzodiazepines, antidepressants, mood stabilizers, anticholinergic agents, and laxatives. Information on diagnoses made by the consulting physician according to the International Classification of Diseases (ICD-10) was also extracted.

All procedures involving human subjects/patients were approved by the RWTH-Aachen University regulatory authority.

#### Lausanne cohort

The dataset was based on two longitudinal cohort studies, the PsyMetab and the PsyClin studies, including 174 in- and outpatients treated with clozapine collected in the Department of Psychiatry of the Lausanne University Hospital, Switzerland. The following data on all patients were extracted from the existing database: age, sex, BMI, smoker status, clozapine dosing regimen, clozapine daily dose as well as concomitant use of other antipsychotics, benzodiazepines, antidepressants, mood stabilizers, anticholinergic agents, and laxatives. Information on diagnoses made by the consulting physician according to the International Classification of Diseases (ICD-10) was also extracted. Data on clinical effectiveness and safety were not available for this dataset. Trough clozapine plasma concentrations were quantified using a previously validated ultra-high-performance liquid chromatography (Waters ACQUITY UPLC I-Class) coupled to electrospray ionization–tandem mass spectrometry [22].

Ethical approval was warranted by the Ethics Committee of the Canton Vaud (CER-VD) for PsyMetab, upon signature of an informed consent for included patients. The CER-VD also granted access to clinical data of patients followed at the Department of Psychiatry of Lausanne University Hospital from 2007 to 2015 (PsyClin) because of the non-interventional post hoc analysis design.

The authors assert that all procedures contributing to this work comply with the ethical standards of the relevant national and institutional committees on human experimentation and with the Helsinki Declaration of 1975, as revised in 2013 [23].

### Statistical analysis

Continuous variables are presented as means with standard deviations, and categorical variables as numbers and percentages. The Shapiro–Wilk test was used to test for normality, and the Levene’s test to assess the homogeneity of variance. Comparisons were performed using the Mann–Whitney *U* test. Categorical variables were analyzed by the Pearson’s Chi-square (or Fisher's exact) test. Given the non-normal distribution of CGI-S ratings, a robust bootstrapping analysis of covariate (ANCOVA) was conducted to check on potential differences in CGI-S ratings between patients receiving clozapine once- vs. multiple-daily stratified by different clozapine daily doses. Additionally, we applied generalized logistic regression models to assess the risk of ADRs in total and for UKU subscales including the following co-variates: clozapine regimen (once- vs. multiple-daily), clozapine daily dose, age, sex, BMI, smoker status and clozapine plasma concentration. Post hoc Holm–Bonferroni correction was applied to account for multiple testing. Given the substantial role of antipsychotic co-medication, we performed a sensitivity analysis comparing once- vs. multiple-daily dosing in a sub-cohort receiving clozapine treatment without any other antipsychotic. We also performed a sensitivity analysis including only patients with schizophrenia-spectrum disorders. All analyses were performed with SPSS for Windows 25.0 (Chicago, Illinois) and R [24].

### Systematic review and meta-analysis

The systematic review and meta-analysis were conducted and reported with the use of MOOSE (Meta-analysis of observational studies in epidemiology) guidelines [25] (Supplementary table S2) and registered with PROSPERO (registration number CRD42022300114). Studies comparing different clozapine daily dosing regimens were identified by searching Medline and Embase, using the following search terms: clozapine AND (“divided dosing” OR “split dosing” OR “multiple dosing” OR “once daily” OR “twice daily”). Databases were searched last on February 1, 2022, for publications without language restriction since data inception. References from identified studies were hand-searched for additional studies. Additionally, contacts with known research groups carrying out research on clozapine were performed to identify possible unpublished data.

#### Inclusion and exclusion criteria

##### Type of studies

Included were studies reporting on different clozapine daily dosing regimens. Case reports were excluded.

##### Types of participants

Patients treated with different clozapine daily dosing regimens without restrictions regarding age, sex, diagnosis, treatment setting, illness duration, and dosage or duration of clozapine treatment were included.

##### Comparator

Patients receiving clozapine once-daily versus multiple doses daily.

##### Types of exposure

Antipsychotic medication with clozapine.

##### Outcomes

The primary outcome was defined as standardized mean difference (SMD) of clinical symptom severity assessment scales ratings between patients treated with clozapine once- vs. multiple-daily dosing. Further, we estimated a pooled odds ratio (OR) for ADRs; ADRs were defined on the basis of description (binary) [26], or, in cases of standardized scales, such as the Glasgow Antipsychotic Side-Effect Scale for Clozapine well-established thresholds were used [14, 27]. Meta-analyses also included differences for age, percentage males, percentage smokers, clozapine daily dose, co-medication with other antipsychotics, benzodiazepines, antidepressants, mood stabilizers, anticholinergics, and laxatives between patients receiving clozapine once- vs. multiple-daily dosing.

Selection of eligible studies was independently performed by two authors (DMH and GS). In case of doubt, papers were discussed, and consensus was reached. As consensus was reached in all cases, no additional co-author was involved.

##### Data extraction

Two authors (DMH and GS) independently extracted data regarding sample sizes, demographic characteristics, psychopathological ratings, daily clozapine dosages and clozapine dosing regimens, concomitant medication, and ADRs. When data were not provided, authors were contacted.

##### Quality of studies

We assessed the quality of studies contributing to the primary outcome; the modified version of the Newcastle–Ottawa scale for cross-sectional studies was used for quality assessment [28]; we removed the item “representativeness of the exposed cohort”, that we judged to be related to applicability, and added ascertainment of clozapine treatment by assessment of clozapine plasma or serum levels.

##### Statistical analysis

For the meta-analysis, a random-effects model for outcomes was used, given the potential heterogeneity related to patient populations, treatment settings, and the inherently large variability of the variables. Results were summarized using SMD and 95% confidence intervals (95%CI) and were presented in Forest plots. The heterogeneity variance parameter (*τ*^2^) was calculated using the DerSimonian–Laird estimator [29]. When more than one cohort was reported in one study, they were treated separately. We also estimated a pooled OR for ADRs using a random-effects model. We calculated the I-square (*I*^2^) statistic as a measure of the proportion of variability that can be attributed to heterogeneity [30]. Mean differences (MD, 95%CI) were estimated for age and clozapine daily dose, whereas ORs (95%CI) were estimated for percentage of males, percentage of smokers, co-medication with other antipsychotics, benzodiazepines, antidepressants, mood stabilizers, anticholinergics, and laxatives. Further, a sensitivity analysis excluding low-quality studies was conducted. Last, we examined the potential of publication bias using funnel plots and Egger’s test [31]. All analyses were performed using the meta package in R [24].

## Results

### AGATE dataset

Details for clozapine dosing regimen were available for a total of 1494 patients, who were included in our analysis. Patients’ demographic and clinical characteristics and details on psychiatric diagnoses are summarized in the Table 1. Clozapine was prescribed multiple-daily in approximately three-fourths of patients (*n* = 1117, 74.8%). Patients on multiple-daily dosing of clozapine received almost twice higher mean daily doses of clozapine compared to once-daily dosing (*p* < 0.001, Table 1) resulting in 1.5-fold higher mean trough clozapine levels (*p* < 0.001, Table 1).

**Table 1:** Differences in patient demographic and clinical characteristics between clozapine once- and multiple-daily dosing patients

	AGATE dataset			Lausanne dataset		
	Once-daily (*n* = 377)	Multiple-daily (*n* = 1117)	*p*- value	Once-daily (*n* = 56)	Multiple-daily (*n* = 118)	*p*- value
^a^Age, years, mean ± SD	42.1 ± 14.7	44.0 ± 15.1	**0.045**	53.3 ± 19.3	52.6 ± 20.1	0.88
Sex, male, *n* (%)	238 (63.1)	687 (61.5)	0.57	27 (48.2%)	54 (45.8%)	0.87
^b^Body mass index (kg/m^2^), mean ± SD	28.3 ± 6.0	28.4 ± 5.9	0.85	26.78 ± 5.54	25.89 ± 5.13	0.43
^c^Smokers, *n* (%)	155 (52.0)	538 (57.4)	0.11	21 (37.5%)	43 (36.4%)	0.73
Clozapine daily dose, mg/day, mean ± SD	193.6 ± 111.3	364.2 ± 168.7	**0.001**	145.0 ± 126.0	268.0 ± 193.7	**0.001**
^d^Clozapine plasma concentration (ng/mL), mean ± SD	257.4 ± 199.9	383.2 ± 236.7	**0.001**	197.3 ± 180.2	313.5 ± 228.5	**0.003**
^d^C/D ratio (ng/mL/mg/day), mean ± SD	1.5 ± 1.3	1.2 ± 0.9	**0.001**	1.67 ± 1.5	1.43 ± 1.55	0.13
^e^Concomitant medications						
Other antipsychotics, *n* (%)	180 (47.7)	701 (62.8)	**0.001**	17 (30.4%)	34 (28.8%)	0.86
Benzodiazepines, *n* (%)	60 (15.9)	249 (22.3)	**0.008**	22 (39.3%)	68 (57.6%)	**0.034**
Mood stabilizers, *n* (%)	64 (17.0)	240 (21.5)	0.06	10 (17.9%)	27 (22.9%)	0.55
Antidepressants, *n* (%)	113 (30.0)	230 (20.6)	**0.001**	19 (33.9%)	61 (51.7%)	**0.034**
Anticholinergics, *n* (%)	23 (6.1)	76 (6.8)	0.63	5 (8.9%)	34 (28.8%)	**0.003**
Laxatives, *n* (%)	11 (2.9)	75 (6.7)	**0.006**	12 (21.4%)	40 (33.9%)	0.11
^f^CGI-S, mean ± SD	5.0 ± 0.8	5.1 ± 0.9	0.25	NA	NA	NA
^f^Responders (GCI-I ≥ 2), *n* (%)	31 (13.3)	90 (12.2)	0.65	NA	NA	NA
^f^Responders (GCI-I ≥ 3), *n* (%)	79 (33.3)	283 (37.4)	0.28	NA	NA	NA
^g^Side effects, *n* (%)	34 (17.3)	127 (19.0)	0.58	NA	NA	NA
^g^UKU side effects rating scale						
Psychic, *n* (%)	9 (4.6)	26 (3.9)	0.67	NA	NA	NA
Neurologic, *n* (%)	10 (5.1)	37 (5.5)	0.80	NA	NA	NA
Autonomic, *n* (%)	15 (7.6)	51 (7.6)	0.99	NA	NA	NA
Other, *n* (%)	8 (4.1)	11 (1.6)	**0.042**	NA	NA	NA
^h^Diagnoses according to ICD-10						
Schizophrenia spectrum disorders (F20–29) except for schizoaffective disorder, *n* (%)	217 (83.1)	690 (84.9)	0.07	25 (48.9)	53 (51.4)	0.45
Schizoaffective disorder (F25), *n* (%)	11 (4.2)	50 (6.2)		5 (10.2)	16 (15.5)	
Bipolar disorders (F31), *n* (%)	7 (2.7)	11 (11.4)		5 (10.2)	5 (4.8)	
Depression (F32-F33), *n* (%)	10 (3.8)	12 (1.5)		7 (14.3)	6 (5.1)	
Other	16 (6.1)	50 (6.2)		8 (16.3)	22 (21.3)	

Numbers in bold indicate significant differences after Holm–Bonferroni multiple comparison correction

*AGATE* Arbeitsgemeinschaft Arzneimitteltherapie bei psychiatrischen Erkrankungen; *CGI-S* Clinical Global Impression – Severity; *C/D ratio* concentration-to-dose ratio; *ICD-10* International Classification of Diseases; *NA* not available; *SD* standard deviation; *UKU* Udvalg for kliniske undersogelser

^a^Data was missing for 23 (1.5%) patients in AGATE Dataset

^b^Data was missing for 223 (14.9%) patients in AGATE Dataset and 21 (12.1%) in Lausanne dataset

^c^Data was missing for 258 (17.3%) patients in AGATE Dataset and 23 (13.2%) in Lausanne Dataset

^d^Data was missing for 43 (24.7%) patients in Lausanne Dataset

^e^Data was missing for 25 (14.4%) patients in Lausanne Dataset

^f^Data was missing for 501 (33.5%) patients in AGATE Dataset

^g^Data was missing for 629 (42.1%) patients in AGATE Dataset

^h^Data was missing for 420 (28.1%) patients in AGATE Dataset and for 22 (12.6%) patients in Lausanne Dataset

Patients prescribed multiple-daily dosing received 32% more often co-medication with other antipsychotics (*p* < 0.001), 40% more often benzodiazepines (*p* < 0.008), 31% less often antidepressants (*p* < 0.001), and 2.3 times more often laxatives (*p* < 0.006) compared to patients prescribed clozapine once-daily.

Scores for CGI-S and CGI-I were available in 972 (65.1%) of patients; no differences were reported for the clinical symptom severity or the responders rate between patients treated with multiple- vs. once-daily dosing clozapine (*p* = 0.25 and *p* = 0.65, Table 1). Based on the results of bootstrapping ANCOVA, no differences for CGI-S scores were reported between patients receiving clozapine once vs. multiple-daily when stratifying based on different clozapine daily doses (Supplementary table S3).

Information about ADRs was available in total of 865 (57.9%) patients. There were no differences in the rates of psychic, neurologic, autonomic, or other ADRs between the two groups (Table 1). The odds of experiencing of ADRs (in total) were increased for higher clozapine plasma concentrations (OR [95% CI] = 1.25, 1.04–1.52, *p* = 0.021), whereas the odds of other ADRs were higher in patients with higher BMI values (OR [95% CI] = 1.15, 1.08–1.23, *p* < 0.001) and lower in non-smokers (OR [95% CI] = 0.43, 0.20–0.94, *p* = 0.034) (Supplementary Table S4).

Comparisons of once- vs. multiple-daily dosing in patients receiving clozapine as the only antipsychotic agent are summarized in Supplementary Table S5a; no differences between once- vs. multiple-daily dosing from our main analysis survived in the sensitivity analysis apart from daily dose, plasma concentrations and concentration-to-dose (C/D) ratios of clozapine (*p* = 0.001 in all three comparisons).

Comparisons of once- vs. multiple-daily dosing in patients with schizophrenia-spectrum disorders are summarized in Supplementary Table S5b; except for age (*p* = 0.08) and co-medication with laxatives (*p* = 0.05), differences for daily doses of clozapine (*p* < 0.001), plasma concentrations of clozapine (*p* < 0.001), C/D ratios (*p* < 0.007), and co-medication with other antipsychotics, benzodiazepines and antidepressants (*p* < 0.002, *p* < 0.001 and *p* < 0.001, respectively) survived in the sensitivity analysis.

### Lausanne dataset

A total of 174 patients were included in this dataset. Clozapine was prescribed in multiple-dosing regimens in approximately two-thirds of the patients (*n* = 118, 67.8%). Comparisons of demographic and clinical characteristics of patients receiving clozapine multiple- vs. once-daily dosing are summarized in Table 1. There was no difference between groups regarding age, sex distribution, BMI, or smoker status (*p* = 0.88, *p* = 0.87, *p* = 0.43 and *p* = 0.73 respectively, Table 1). Patients prescribed multiple-daily dosing received 85% higher daily doses of clozapine compared to once-daily dosing (*p* < 0.001), resulting in 59% higher trough clozapine levels (*p* = 0.003). Patients receiving multiple- vs. once-daily dosing clozapine were prescribed 3.2 times more often anticholinergics (*p* = 0.003), and there was a trend in higher prescription rates of benzodiazepines and antidepressants in patients receiving multiple- vs. once-daily dosing (*p* = 0.034 for both comparisons).

In patients receiving clozapine as the only antipsychotic agent, no differences from our main analysis survived apart from daily dose (*p* < 0.001) and plasma concentrations (*p* < 0.009), which were higher in patients treated with multiple- vs. once-daily dosing, whereas prescription of anticholinergic agents and laxatives (*p* = 0.019 and *p* = 0.033), which were both more frequent in the multiple- vs. once-daily dosing (Supplementary Table S5a).

In patients with schizophrenia-spectrum disorders, differences for daily clozapine doses (*p* < 0.003), plasma concentrations of clozapine (*p* < 0.019), and co-medication with laxatives (*p* < 0.003) remained significant in the sensitivity analysis (Supplementary Table S5b).

### Systematic review and meta-analysis

The electronic database search yielded 98 articles from Medline and 548 from Embase and one from the full-text reviewed articles' reference lists. We also included the two previously datasets from AGATE and Lausanne. Following removal of duplicates, 574 unique articles remained and were screened based on title and abstract. Consequently, 552 articles were excluded leading to 23 articles, which were full-text screened. Afterward, 12 papers were excluded due to lack of information on clozapine daily dosing regimen, two papers for reporting pharmacokinetic modeling, one paper not related to clozapine, one review and one paper reporting duplicate data. Ultimately, four studies and our two datasets (AGATE and Lausanne) reporting a total eight cohorts (Table 2) fulfilled all inclusion criteria and were used for data extraction (Supplementary figure S1).

**Table 2:** Characteristics of included studies (in chronological order)

Author, year	Total *n*	Dosing regimen	*n*	Age (SD), years	% *♂*	% Smokers	Clozapine dose (SD), mg/day	Clinical symptoms		% ADR	Co-medication with (%)						Quality
								Scales	Mean scores (SD)		Other AP	BZD	AD	MS	AC	Laxatives	
Takeuchi, 2016, CAMH Cohort	676	Once-daily	508	44.4 (13.0)	71.3	NA	368 (138)	NA	NA	NA	15.2	10.6	23.2	15.0	3.5	NA	–^a^
		Multiple-daily	168	50.4 (11.5)	69.6	NA	489 (151)		NA	NA	20.8	15.7	26.5	29.5	9.6	NA	
Takeuchi, 2016, ZHH Cohort	308	Once-daily	229	41.0 (12.3)	58.5	NA	339 (132)	BPRS	177 (79.7)	NA	19.2	19.2	45.0	27.5	10.0	NA	Good
		Multiple-daily	79	43.4 (11.9)	78.5	NA	465 (164)		62 (80.5)	NA	32.9	40.5	49.4	27.8	22.8	NA	
de Filippis, 2021	21	Once-daily	9	61.4 (5.6)	55.6	44.4	143.1 (70.5)	BPRS	34.7 (7.9)	33.3	22.2	11.1	0.0	22.2	22.2	11.1	Good
		Multiple-daily	12	54.7 (6.7)	91.7	83.3	358.3 (87.5)		32.5 (4.6)	8.3	25.0	16.7	0.0	16.7	8.3	16.7	
Kitagawa, 2021, Yamanashi Cohort	48	Once-daily	12	43.7 (10.4)	58.3	50.0	258 (122)	BE-PSD	10.8 (3.9)	50.0	8.3	25.0	16.7	50.0	0.0	66.7	Good
		Multiple-daily	36	45.7 (9.8)	55.6	41.7	399 (115)		10.2 (4.3)	41.7	11.1	33.3	5.6	63.9	19.4	83.3	
Kitagawa, 2021, Okayama Cohort	60	Once-daily	35	39.3 (10.3)	48.6	31.4	353 (144)		13.2 (3.1)	37.1	14.3	31.4	25.7	77.1	25.7	80.0	Good
		Multiple-daily	25	44.2 (9.4)	68.0	40.0	350 (147)		13.2 (3.6)	37.5	4.0	28.0	16.0	68.0	12.0	84.0	
Leclerc, 2021	29	Once-daily	19	20.6 (2.7)	15.0	NA	347 (136)	CGI-S	2.6 (1.7)	0.0	0.0	10.5	26.3	10.5	0.0	NA	Poor
		Multiple-daily	10	22.1 (3.3)	11.0	NA	403 (163)		3.7 (1.5)	20.0	0.0	0.0	20.0	30.0	0.0	NA	
AGATE Cohort (unpublished)	1494	Once-daily	377	42.1 (14.7)	63.1	52.0	193.1 (111.6)	CGI-S	5.0 (0.8)	17.3	47.7	15.9	30.0	17.0	6.1	2.9	Good
		Multiple-daily	1117	44.0 (15.1)	61.5	57.4	383.2 (236.7)		5.1 (0.9)	19.0	62.8	22.3	20.6	21.5	6.8	6.7	
Lausanne Cohort (unpublished)	174	Once-daily	56	53.3 (19.3)	48.2	37.5	145.0 (126.0)	NA	NA	NA	30.4	39.3	33.9	17.9	8.9	21.4	-^a^
		Multiple-daily	118	52.6 (20.1)	45.8	36.4	268.0 (193.7)		NA	NA	28.8	57.6	51.7	22.9	28.8	33.9	
Total	2,810	Once-daily	1,245	43.1 (14.1)	64.7	50.9	296.2 (153.0)	BE-PSD: 1 BPRS: 2 CGI-S: 2 NA: 2		19.2	26.2	15.8	29.6	20.1	6.4	4.9	Good: 5 Poor: 1
		Multiple-daily	1,565	45.3 (15.2)	62.5	56.5	389.8 (223.4)			19.9	51.4	25.3	24.5	24.5	9.9	12.8	

*♂* males; *AC* anticholinergic agents; *AD* antidepressants; *ADR* adverse drug-induced reactions; *AGATE* Arbeitsgemeinschaft Arzneimitteltherapie bei psychiatrischen Erkrankungen; *AP* antipsychotics; *BE-PSD* Brief Evaluation of Psychosis Symptom Domains; *BPRS* Brief Psychiatric Rating Scale; *BZD* benzodiazepines; *CAMH* Centre for Addiction and Mental Health; *CGI-S* Clinical Global Impression – Severity scale; *MS* mood stabilizers; *NA* not available; *n* number of patients; *SD* standard deviation; *ZHH* Zucker Hillside Hospital

^a^This cohort did not contribute to primary outcome

#### Study and patient characteristics

We meta-analyzed eight cohorts for a total of 2,810 clozapine-treated individuals with 1565 vs. 1245 patients treated with multiple- vs. once-daily dosing regimen, respectively (Table 2). There was no difference for age (*p* = 0.09) or the percentage of sex (*p* = 0.07) between the two groups (Supplementary figure S2A, B). Patients treated with clozapine multiple-dosing were 1.22-fold more often smokers (95%CI: 1.09–1.38, *p* < 0.001, Supplementary figure S2C) and received 126.2 mg more clozapine daily (95%CI: 91.4–161.1, *p* < 0.001, Supplementary figure S2D) compared to patients prescribed clozapine once-daily.

#### Quality assessment

Of the six studies contributing to the primary outcome, five were rated as good, and one as poor quality (Supplementary table S6).

#### Primary outcome

**Fig. 1:**
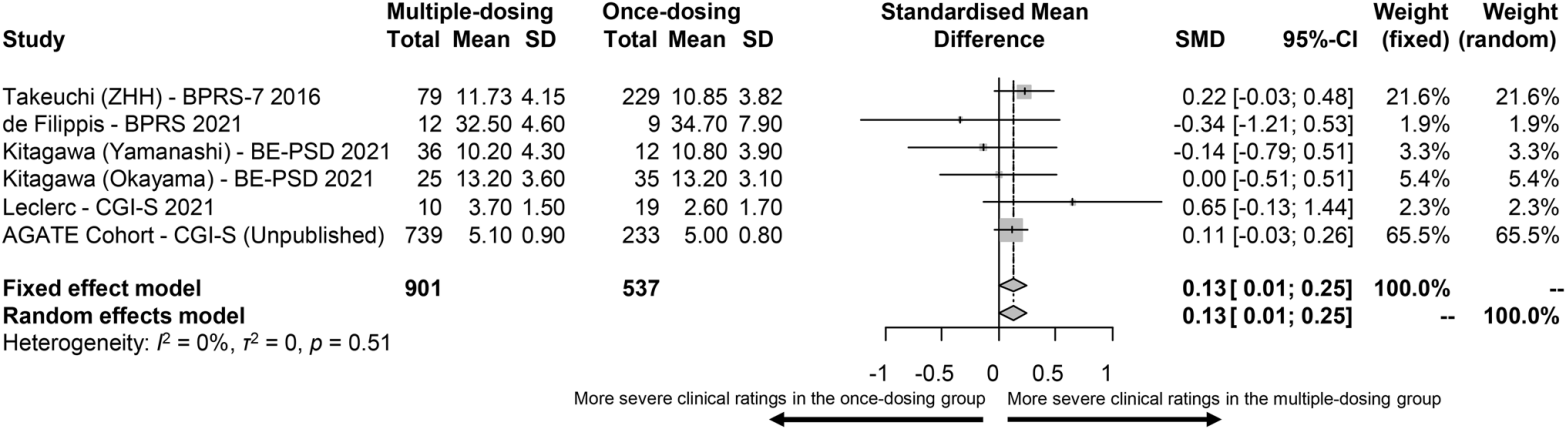
Forest plot for clinical symptom rating scores in patients treated with clozapine multiple- vs. once-daily dosing

**Fig. 2:**
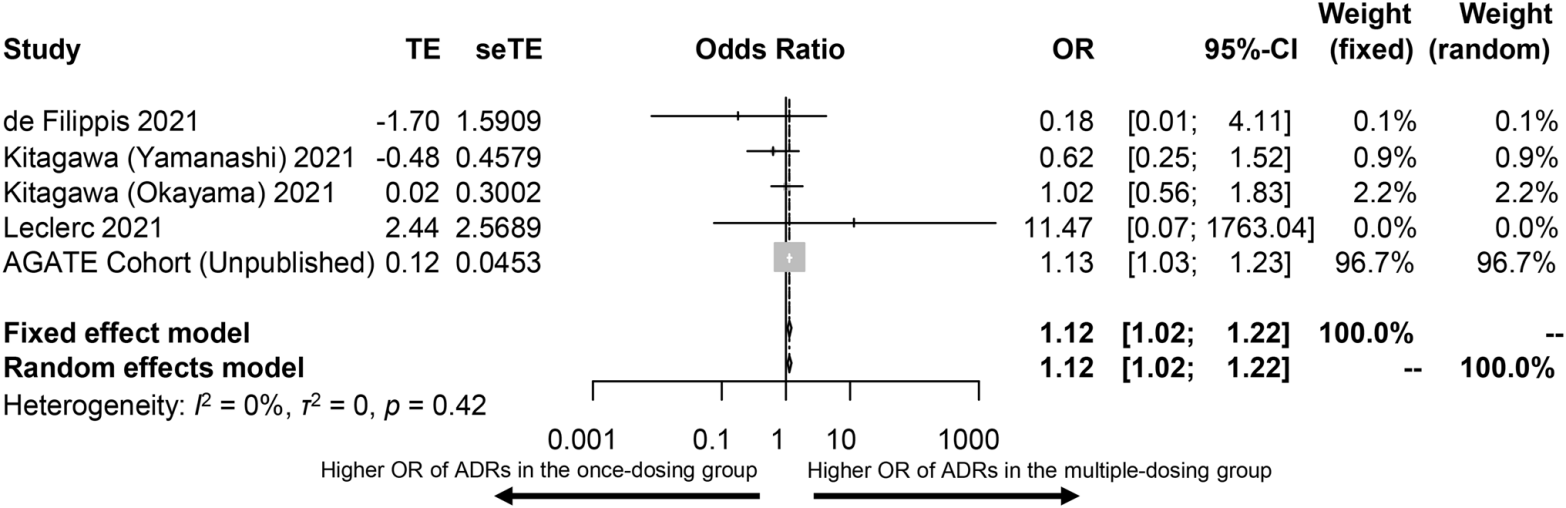
Forest plot for adverse drug-induced reactions (ADR) in patients treated with clozapine multiple- vs. once-daily dosing

In six cohorts (*n* = 1438) including 901 vs. 537 patients receiving clozapine multiple- vs. once-daily dosing, clinical symptom rating scores were higher in patients treated with clozapine multiple- vs. once-daily dosing (SMD = 0.13, 95%CI = 0.01–0.25, *p* = 0.036, Fig. 1). Heterogeneity was minimal (*I*^*2*^ = 0%, *τ*^*2*^ = 0, *p* = 0.51). The OR of clozapine-related ADRs was 1.12 (95%CI = 1.02–1.22, *p* = 0.01, Fig. 2) in 736 patients prescribed clozapine multiple- vs. 263 patients prescribed once-daily dosing.

#### Secondary outcomes

Patients treated with multiple-dosing clozapine were more likely to receive co-medication with other antipsychotics (OR = 1.52, 95%CI = 1.23–1.88, *p* < 0.001, Supplementary figure S3A), benzodiazepines (OR = 1.94, 95%CI = 1.51–2.50, *p* < 0.0001, Supplementary figure S3B), anticholinergics (OR = 1.83, 95%CI = 1.03–3.24, *p* = 0.039, Supplementary figure S3E), and laxatives (OR = 2.16, 95%CI = 1.84–2.55, *p* < 0.001, Supplementary figure S3F). There were no group differences regarding co-medication with antidepressants (OR = 0.92, 95%CI = 0.60–1.42, *p* = 0.72, Supplementary figure S3C) and mood stabilizers (OR = 1.36, 95%CI = 0.98–1.88, *p* = 0.064, Supplementary figure S3D).

#### Sensitivity analysis

In a sensitivity analysis excluding the one study rated as of poor quality, we did not report differences for clinical symptom rating scores between patients receiving clozapine multiple- vs. once-daily dosing (SMD = 0.11, 95%CI =  – 0.006–0.24, *k* = 5, *n* = 1,409, *p* = 0.06) with heterogeneity being minimal (*I*^*2*^ = 0%, *τ*^*2*^ = 0.0), whereas we estimated an OR of clozapine-related ADRs of 1.10 (95%CI = 0.96–1.26, *k* = 4, *n* = 970, *p* = 0.15) in patients prescribed clozapine multiple- vs. once-daily dosing with heterogeneity remaining low (*I*^2^ = 2.9%, *τ*^2^ = 0.003).

#### Publication bias

Neither the visual inspection of funnel plots (Supplementary figure S4) nor Egger’s test results (*p* = 0.77) revealed any signs of publication bias.

## Discussion

We used two complementary approaches to assess differences for effectiveness and safety of clozapine once- vs. multiple-daily dosing in two European hospital networks, but also in several clinical settings from around the world. Despite differences regarding clozapine prescription guidelines and patterns worldwide, our primary findings aligned with our meta-analytical evidence to a substantial extend.

### Primary data

Dividing clozapine daily dose is recommended by manufacturer due to its relatively short half-life time aiming to potentially improve clozapine’s tolerability and efficacy [32]. However, our findings in two Central European hospitals did not suggest any tolerability differences between patients receiving once- vs. multiple-dosing of clozapine. Nevertheless, when assessing indirect measures of tolerability, co-medication with laxatives in the AGATE cohort and co-medication with anticholinergic agents in the Lausanne cohort was more frequent in patients treated with multiple- vs. once-daily dosing. Therefore, dividing clozapine dose over the day was less useful regarding the risk of some ADRs types, such as constipation and extrapyramidal symptoms. On the other hand, the risk of the ADRs needs to be considered also under the light of the higher frequency of co-medication with other antipsychotics (apart from clozapine) for patients with multiple- vs. once-daily dosing in the AGATE cohort. For example, in case of extrapyramidal symptoms, for which we assessed the co-medication with anticholinergic agents as surrogate, mechanisms are less likely to include clozapine [8], but rather the prescription of other antipsychotic agents. Interestingly, in our logistic regression, higher clozapine concentrations were associated with higher odds of experiencing ADRs, whereas daily doses did not; in other words, clozapine plasma concentrations may be better predictors of ADRs risk over clozapine daily dose. Moreover, the higher clozapine daily doses leading to higher clozapine levels may be also part of the mechanism, underlying worse tolerability profiles in patients with multiple-daily dosing [6]. The role of elevated clozapine levels in some types of clozapine-related ADRs including hyper-salivation has been previously highlighted [33, 34]. Last, clozapine dose and age have been previously reported as independent predictors of laxative use in clozapine-treated patients [35].

In Germany and Switzerland, splitting of the clozapine maintenance dose over the day is generally recommended, with doses < 200 mg/d recommended once-daily at afternoon [32]. The guidelines of the German Association for Psychiatry, Psychotherapy, Psychosomatics and Neurology (Deutsche Gesellschaft für Psychiatrie und Psychotherapie, Psychosomatik und Nervenheilkunde: DGPPN) however suggest the minimal effective daily dose of clozapine for TRS treatment to be 300 mg/day [36]. This fact could explain that the majority of patients in our cohorts were treated with multiple-daily dosing regimens contrasting prescription trends in the US and Canada [13]. Given that recommendations do not essentially differ among countries, we hypothesize that gaps in clozapine dosing regimens might be associated with differences in clinicians’ attitudes.

### Meta-analytical data

Our findings suggested slightly better tolerability in patients with once- vs. multiple-daily dosing; in fact, the risk of ADRs was 12% higher in patients with multiple- vs. once-daily dosing regimens, although this did not survive in our sensitivity analysis. Among surrogates of tolerability, in patients prescribed clozapine multiple-daily dosing, the prescription of anticholinergic agents and laxatives was approximately twice more frequent than in patients with once-daily dosing. As in our primary data, differences regarding tolerability need to be interpreted in light of potential interactions with the more frequently co-medication with other antipsychotics as well.

It is also particularly important that patients in the multiple-dosing group had more severe clinical symptoms and were 50% more likely to receive co-medication with other antipsychotics. We assume that patients responding less well to clozapine may end up receiving higher clozapine daily doses frequently augmented with other antipsychotics. Following the manufacturer recommendations, clinicians split high clozapine daily doses over the day. From another side, there was a trend for patients prescribed clozapine multiple-daily dosing regimens, being older than patients with once-daily. The delayed initiation of clozapine medication may worsen the treatment results in TRS [37–40], and the meta-analysis performed by Okhuijsen-Pfeifer et al. suggested, that clozapine may be more effective when used at the initial time point of the illness [41]. Moreover, multiple-daily dosing was more frequent among smokers; clinicians might have aimed to mitigate inducing effects of smoking on clozapine bioavailability and efficacy [42].

Ultimately, patients prescribed clozapine multiple-daily dosing may represent a patient subgroup that may respond less well to clozapine, leading to combination with other antipsychotics and most likely to a higher risk of ADRs.

### Limitations

The cross-sectional design of our study as well as of the studies included in our meta-analysis does not allow any hypotheses on causal mechanisms underlying our findings. We also acknowledge the complexity of concluding upon the tolerability and efficacy given the very heterogeneous profile of the patients prescribed clozapine once- vs. multiple-daily dosing. Data for potentially crucial parameters, including onset and duration of illness and clozapine treatment as well as the duration of observation intervals, were not available and could not be included in our primary or meta-analysis. Availability of multiple TDM measurements and clinical ratings per patient could have allowed the use of sophisticated modeling techniques such as causal inference. Physicians’ preferences for splitting (or not) higher doses of clozapine also depending on attention to product monograph recommendation [13] and local guidelines and preferences might have posed some bias to our findings. The side effects' rates in the AGATE dataset were relatively low, which might suggest some under-reporting; however, it is unlikely that this type of bias might account for our findings, as both study groups might have been exposed to it. Our findings may be, at least partially, biased by higher degree of nonadherence among multiple-dosed patients underlying limited effectiveness, and subsequently prescription of higher clozapine daily doses and/or multiple drugs (polypharmacy), potentially increasing the occurrence of ADRs. The use of diagnosis-specific rating scales might have yielded more specificity in our findings, whereas interrater reliability was not assessed. Although clinicians were clearly instructed to draw trough blood, practical challenges in the clinical setting might have led to some deviation in sampling time, accounting for some heterogeneity in the TDM findings, reflecting what happens in daily clinical practice*.* Last, the lack of sophisticated pharmacokinetic data including trough and peak clozapine levels in our two datasets did not allow for testing of smaller intra-individual variations for clozapine levels in patients with multiple- vs. once-daily clozapine dosing suggested by pharmacokinetic simulations [6].

In conclusion, patients on multiple-daily clozapine regimens comprise a patient subgroup that responds less well to clozapine, being prescribed higher daily doses of clozapine and more frequently co-medication with other antipsychotics, benzodiazepines, anticholinergic agents, and laxatives. Dividing clozapine dose over the day may not reduce the ADRs burden, although polypharmacy rather than clozapine might be underlying worse tolerability profiles. At this stage, as data do not allow strong conclusions, the dosing selection decision should be individualized based on available evidence, and in considering potential advantages in terms of treatment adherence and patients’ preference; for example, patients where once-daily dosing may have a strong positive impact on adherence, such as patients living in the community with daily supervision of adherence through Assertive Community Treatment (ACT) teams. Prospective studies using specific designs will provide valuable insight in pathways linking selection of dosing regimens, clinical outcomes, and safety profiles. For example, a randomized clinical trial of different clozapine dosing regimens has not been available yet.

## Supplementary Information

Below is the link to the electronic supplementary material.

Supplementary file1 (DOCX 271 KB)

Supplementary file2 (PDF 7344 KB)

Supplementary file3 (PDF 7609 KB)

Supplementary file4 (PDF 245 KB)

## Data Availability

Primary data are available upon reasonable request.
