# Supplementary material for: Clozapine once- versus multiple-daily dosing: a two-center cross-sectional study, systematic review and meta-analysis
Source: Eur Arch Psychiatry Clin Neurosci. 2022 Dec 29;273(7):1567–78. doi: 10.1007/s00406-022-01542-1 (PMC10465369; doi:10.1007/s00406-022-01542-1)
Supplement: Supplementary file 1 — Supplementary file1 (DOCX 271 KB) [file 406_2022_1542_MOESM1_ESM.docx]

**Clozapine Once- Versus Multiple-Daily Dosing: a two-center cross-sectional study,**

**systematic review and meta-analysis**

**Supplementary**

**Supplementary material**

**Contents**

[**Supplementary table S1:** STROBE Statement Checklist 3](#_Toc117233185)

[**Supplementary table S2:** MOOSE Checklist 6](#_Toc117233186)

[**Supplementary figure S1.** MOOSE Checklist Study Flow Diagram 7](#_Toc117233187)

[**Supplementary figure S2:** Forest plots for age, sex, smoking status, and clozapine daily dose 8](#_Toc117233189)

[**Supplementary figure S3:** Forest plots for co-medication 9](#_Toc117233190)

[**Supplementary figure S4:** Funnel plot 10](#_Toc117233191)

[**Supplementary table S3.** Differences in CGI-S ratings stratified by different clozapine daily doses 13](#_Toc117233192)

[**Supplementary table S4.** Odds ratios of adverse drug reactions 14](#_Toc117233193)

[**Supplementary table S5a.** Sensitivity analysis in patients receiving clozapine as the only antipsychotic agent. 15](#_Toc117233194)

[**Supplementary table S5b.** Sensitivity analysis including only patients with schizophrenia-spectrum disorders. 16](#_Toc117233195)

[**Supplementary table S6:** Quality assessment of included cohorts 17](#_Toc117233196)

#

# **Supplementary table S1:** STROBE (Strengthening the reporting of observational studies in epidemiology) Statement Checklist

|  | Item No. | Recommendation | Page  No. |
| --- | --- | --- | --- |
| **Title and abstract** | 1 | (*a*) Indicate the study’s design with a commonly used term in the title or the abstract | 1 |
|  |  | (*b*) Provide in the abstract an informative and balanced summary of what was done and what was found | 3 |
| Introduction | | | |
| Background/ rationale | 2 | Explain the scientific background and rationale for the investigation being reported | 6 |
| Objectives | 3 | State specific objectives, including any prespecified hypotheses | NA |
| Methods | | | |
| Study design | 4 | Present key elements of study design early in the paper | 7-8 |
| Setting | 5 | Describe the setting, locations, and relevant dates, including periods of recruitment, exposure, follow-up, and data collection | 7-8 |
| Participants | 6 | Give the eligibility criteria, and the sources and methods of selection of participants | 7-8 |
|  |  | For matched studies, give matching criteria and the number of controls per case | NA |
| Variables | 7 | Clearly define all outcomes, exposures, predictors, potential confounders, and effect modifiers. Give diagnostic criteria, if applicable | 7-8 |
| Data sources/ measurement | 8* | For each variable of interest, give sources of data and details of methods of assessment (measurement). Describe comparability of assessment methods if there is more than one group | 7-8 |
| Bias | 9 | Describe any efforts to address potential sources of bias | NA |
| Study size | 10 | Explain how the study size was arrived at | NA |

Continued on next page

| Quantitative variables | 11 | Explain how quantitative variables were handled in the analyses. If applicable, describe which groupings were chosen and why | 7-8 |
| --- | --- | --- | --- |
| Statistical methods | 12 | (*a*) Describe all statistical methods, including those used to control for confounding | 8 |
|  |  | (*b*) Describe any methods used to examine subgroups and interactions | 8 |
|  |  | (*c*) Explain how missing data were addressed | NA |
|  |  | If applicable, describe analytical methods taking account of sampling strategy | NA |
|  |  | (*d*) Describe any sensitivity analyses | NA |
| Results | | | |
| Participants | 13 | (a) Report numbers of individuals at each stage of study—eg numbers potentially eligible, examined for eligibility, confirmed eligible, included in the study, completing follow-up, and analysed | 10-11, Table 1 |
|  |  | (b) Give reasons for non-participation at each stage | NA |
|  |  | (c) Consider use of a flow diagram | NA |
| Descriptive data | 14 | (a) Give characteristics of study participants (eg demographic, clinical, social) and information on exposures and potential confounders | Table 1 |
|  |  | (b) Indicate number of participants with missing data for each variable of interest | Table 1 |
| Outcome data | 15 | Report numbers in each exposure category, or summary measures of exposure | Table 1 |
| Main results | 16 | (*a*) Give unadjusted estimates and, if applicable, confounder-adjusted estimates and their precision (eg, 95% confidence interval). Make clear which confounders were adjusted for and why they were included | Table 1 |
|  |  | (*b*) Report category boundaries when continuous variables were categorized | NA |
|  |  | (*c*) If relevant, consider translating estimates of relative risk into absolute risk for a meaningful time period | NA |

Continued on next page

| Other analyses | 17 | Report other analyses done—eg analyses of subgroups and interactions, and sensitivity analyses | Table 1 |
| --- | --- | --- | --- |
| Discussion | | | |
| Key results | 18 | Summarise key results with reference to study objectives | 13 |
| Limitations | 19 | Discuss limitations of the study, taking into account sources of potential bias or imprecision. Discuss both direction and magnitude of any potential bias | 14 |
| Interpretation | 20 | Give a cautious overall interpretation of results considering objectives, limitations, multiplicity of analyses, results from similar studies, and other relevant evidence | 14 |
| Generalisability | 21 | Discuss the generalisability (external validity) of the study results | 14 |
| Other information | |  | |
| Funding | 22 | Give the source of funding and the role of the funders for the present study and, if applicable, for the original study on which the present article is based | 2 |

# **Supplementary table S2:** MOOSE (Meta-analyses Of Observational Studies in Epidemiology) Checklist

Data available as a separate PDF-File.

# **Supplementary figure** **S1.** MOOSE Checklist Study Flow Diagram

Full-text articles excluded with reason: lack of information on dosing scheme (n = 12), pharmacokinetic modelling (n = 2), not clozapine-related (n = 1), review (n = 1), duplicate (n = 1)

Records identified through database searching
(Medline: n = 98, Embase: n = 548) 02)

Records screened after removal of duplicates
(n = 574)

Full-text articles assessed for eligibility
(n = 23)

Articles excluded based on title and abstract

(n = 551)

Screening

Included

Eligibility

Identification

Identified from full-text articles references (n = 1)

Previously unpublished data

(n = 2)

Studies included in the systematic review and meta-analysis
(n = 6 [8 cohorts])

# **Supplementary figure S2:** Forest plots for age (A), sex (B), smoking status (C), and clozapine daily dose (D)

Figure available as a separate PDF-File.

# **Supplementary figure S3:** Forest plots for co-medication with other antipsychotics (A), benzodiazepines (B), antidepressants (C), mood stabilizers (D), anticholinergics (E), and laxatives (F)

Figure available as a separate PDF-File.

# **Supplementary figure S4:** Funnel plot

**Reference list of published studies included in meta-analysis**

H. Takeuchi *et al.*, “Clozapine administration in clinical practice: once-daily versus divided dosing,” *Acta Psychiatrica Scandinavica*, vol. 134, no. 3, pp. 234–240, Sep. 2016, doi: 10.1111/ACPS.12593.

L. D. Leclerc *et al.*, “A Chart Audit Study of Clozapine Utilization in Early Psychosis,” *J Clin Psychopharmacol*, vol. 41, no. 3, pp. 275–280, May 2021, doi: 10.1097/JCP.0000000000001384.

R. de Filippis *et al.*, “Clozapine Management in Schizophrenia Inpatients: A 5-Year Prospective Observational Study of Its Safety and Tolerability Profile,” *Neuropsychiatr Dis Treat*, vol. 17, pp. 2141–2150, 2021, doi: 10.2147/NDT.S312095.

K. Kitagawa *et al.*, “Clozapine Once-Daily Versus Divided Dosing Regimen A Cross-sectional Study in Japan at 2 psychiatric hospitals in,” *Journal of Clinical Psychopharmacology*, vol. 00, 2021, doi: 10.1097/JCP.0000000000001492.

# **Supplementary table S3.** Differences in CGI-S ratings in patients of AGATE cohort receiving clozapine once vs. multiple-daily stratified by different clozapine daily doses

| **Clozapine daily dose** | **n once-daily** | **n multiple-daily** | **CGI-S** **Difference** | **Lower CI** | **Upper CI** | **p-value** |
| --- | --- | --- | --- | --- | --- | --- |
| 25 mg | 94 | 132 | -0.01 | -0.62 | 0.60 | 0.89 |
| 225 mg | 447 | 228 | 0.09 | -0.473 | 0.65 | 0.33 |
| 300 mg | 519 | 150 | 0.18 | -0.43 | 0.79 | 0.07 |
| 400 mg | 477 | 69 | 0.07 | -0.81 | 0.96 | 0.60 |
| 575 mg | 229 | 12 | -0.38 | -1.82 | 1.06 | 0.12 |

AGATE: Arbeitsgemeinschaft Arzneimitteltherapie bei psychiatrischen Erkrankungen; CGI-S: Clinical Global Impression – Severity; CI: 95% confidence interval

The table indicates comparisons of CGI-S ratings between once- vs. multiple-daily dosing in randomly selected groups of patients stratified by the same median clozapine daily doses.

# **Supplementary table S4.** Odds ratios (95% confidence intervals) of adverse drug reactions (ADR) in total and the UKU-subscales in the AGATE cohort

|  | **Total ADRs** | **Psychic ADRs** | **Neurologic ADRs** | **Autonomic ADRs** | **Other ADRs** |
| --- | --- | --- | --- | --- | --- |
| Clozapine once- vs. multiple daily | 1.07 (0.65-1.77) | 0.89 (0.35-2.27) | 0.56 (0.24-1.27) | 1.10 (0.52-2.33) | 0.33 (0.10-1.12) |
| 200mg clozapine daily dose increase | 1.01 (0.56-1.82) | 1.01 (0.78-1.31) | 1.21 (0.98-1.50) | 1.02 (0.84-1.22) | 1.02 (0.70-1.49) |
| Age (years) | 0.96 (0.86-1-07) | 0.90 (0.75-1.09) | 1.22 (0.98-1.52) | 0.93 (0.8-1.08) | 0.84 (0.66-1.07) |
| Sex | 0.79 (0.53-1.18) | 1.59 (0.77-3.32) | 1.42 (0.74-2.70) | 0.68 (0.37-1.25) | 0.54 (0.17-1.73) |
| BMI (kg/m^2^) | 1.02 (0.99-1.05) | 1.01 (0.95-1.07) | 0.99 (0.93-1.04) | 1.00 (0.96-1.05) | **1.15 (1.08-1.23)** |
| Smoker status | 0.82 (0.60-1.12) | 0.87 (0.47-1.58) | 0.66 (0.40-1.06) | 1.02 (0.63-1.63) | **0.43 (0.20-0.94)** |
| 200 ng/mL clozapine plasma concentration increase | **1.25 (1.04-1.52)** | 0.99 (0.71-1.38) | 1.21 (0.98-1.50) | 1.18 (0.96-1.44) | 0.77 (0.43-1.38) |

ADRs: adverse drug reactions; BMI: body mass index; UKU: Udvalg for kliniske undersogelser

Bold number indicates significant predictors. Data was missing for 629 (42.1%) patients in AGATE Dataset.

# **Supplementary table S5a.** Sensitivity analysis in patients receiving clozapine as the only antipsychotic agent.

|  | **AGATE Dataset** | | | **Lausanne Dataset** | | |
| --- | --- | --- | --- | --- | --- | --- |
|  | **Once-daily**  **(n = 197)** | **Multiple-daily**  **(n = 416)** | ***p*- value** | **Once-daily**  **(n = 39)** | **Multiple-daily**  **(n = 84)** | ***p*- value** |
| ^a^Age, years, mean ± SD | 43.1 ± 15.1 | 45.8 ± 15.7 | 0.15 | 53.3 ± 19.6 | 53.0 ± 19.7 | 0.94 |
| Sex, male, n (%) | 134 (68.0) | 239 (57.5) | 0.012 | 17 (43.6) | 39 (46.4) | 0.85 |
| ^b^Body mass index (kg/m^2^), mean ± SD | 27.6 ± 5.7 | 28.4 ± 6.0 | 0.30 | 26.4 ± 5.6 | 26.5 ± 5.3 | 0.68 |
| ^c^Smokers, n (%) | 73 (50.2) | 175 (51.2) | 0.87 | 14 (40.0) | 30 (42.9) | 0.84 |
| Clozapine daily dose, mg/day, mean ± SD | 186.6 ± 107.8 | 328.2 ± 158.5 | **0.001** | 159.2 ± 116.6 | 271.1 ± 176.0 | **0.001** |
| ^d^Clozapine plasma concentration (ng/mL), mean ± SD | 247.2 ± 193.0 | 368.0 ± 246.9 | **0.001** | 197.7 ± 159.9 | 303.5 ± 205.5 | **0.009** |
| ^d^C/D ratio (ng/mL/mg/day), mean ± SD | 1.5 ± 1.2 | 1.3 ± 1.0 | **0.001** | 1.4 ± 1.0 | 1.5 ± 1.6 | 0.38 |
| ^e^Concomitant medications |  |  |  |  |  |  |
| Benzodiazepines, n (%) | 15 (7.6) | 56 (13.5) | 0.035 | 15 (38.5) | 45 (53.6) | 0.13 |
| Mood stabilizers, n (%) | 33 (16.8) | 69 (16.6) | 0.96 | 6 (15.4) | 20 (23.8) | 0.35 |
| Antidepressants, n (%) | 59 (29.9) | 89 (21.4) | 0.021 | 16 (41.0) | 39 (46.4) | 0.7 |
| Anticholinergics, n (%) | 3 (1.5) | 6 (1.4) | 0.94 | 2 (5.1) | 19 (22.6) | **0.019** |
| Laxatives, n (%) | 3 (1.5) | 24 (5.8) | 0.017 | 6 (15.4) | 29 (34.5) | **0.033** |
| ^f^CGI-S, mean ± SD | 4.85 ± 0.9 | 5.1 ± 0.9 | 0.59 | NA | NA | NA |
| ^f^Responders (GCI-I≥2), n (%) | 14 (12.5) | 49 (17.6) | 0.22 | NA | NA | NA |
| ^f^Responders (GCI-I≥3), n (%) | 37 (33.0) | 118 (42.3) | 0.09 | NA | NA | NA |
| ^g^Side effects, n (%) | 15 (15.6) | 38 (15.6) | 1.00 | NA | NA | NA |
| ^g^UKU side effects rating scale |  |  |  | NA | NA | NA |
| Psychic, n (%) | 2 (2.1) | 7 (2.9) | 0.68 | NA | NA | NA |
| Neurologic, n (%) | 5 (5.2) | 6 (2.5) | 0.20 | NA | NA | NA |
| Autonomic, n (%) | 6 (6.3) | 16 (6.6) | 0.91 | NA | NA | NA |
| Other, n (%) | 3 (3.1) | 6 (2.5) | 0.74 | NA | NA | NA |

Bold number indicates significant difference after Holm-Bonferroni multiple comparison correction.

AGATE: Arbeitsgemeinschaft Arzneimitteltherapie bei psychiatrischen Erkrankungen; CGI-S: Clinical Global Impression – Severity; C/D ratio – concentration to dose ratio; NA: not available; SD: standard deviation, UKU: Udvalg for kliniske undersogelser.

^a^Data was missing for 9 (1.5%) patients in AGATE Dataset

^b^Data was missing for 115 (18.8%) patients in AGATE Dataset and 13 (11%) in Lausanne dataset.

^с^Data was missing for 126 (20.6%) patients in AGATE Dataset and 18 (15%) in Lausanne Dataset.

^d^Data was missing for 27 (22%) patients in Lausanne Dataset.

^e^Data was missing for 21 (17%) patients in Lausanne Dataset.

^f^Data was missing for 222 (36.2%) patients in AGATE Dataset.

^g^Data was missing for 274 (44.7%) patients in AGATE Dataset.

# **Supplementary table S5b.** Sensitivity analysis including only patients with schizophrenia-spectrum disorders.

|  | **AGATE Dataset** | | | **Lausanne Dataset** | | |
| --- | --- | --- | --- | --- | --- | --- |
|  | **Once-daily**  **(n = 217)** | **Multiple-daily**  **(n = 690)** | ***p*- value** | **Once-daily**  **(n = 25)** | **Multiple-daily**  **(n = 53)** | ***p*- value** |
| ^a^Age, years, mean ± SD | 40.6 ± 14.0 | 42.7 ± 14.7 | 0.08 | 44.2 ± 14.8 | 48.0 ± 20.1 | 0.45 |
| Sex, male, n (%) | 137 (63.1) | 447 (64.8) | 0.66 | 13 (52.0) | 20 (37.7) | 0.33 |
| ^b^Body mass index (kg/m^2^), mean ± SD | 28.3 ± 5.8 | 28.2 ± 5.7 | 0.82 | 27.4 ± 4.7 | 25.5 ± 5.0 | 0.12 |
| ^c^Smokers, n (%) | 99 (56.6) | 370 (60.6) | 0.34 | 13 (54.2) | 21 (44.7) | 0.47 |
| Clozapine daily dose, mg/day, mean ± SD | 213.5 ± 112.2 | 384.3 ± 168.8 | **0.001** | 207.0 ± 142.8 | 316.7 ± 162.5 | **0.003** |
| ^d^Clozapine plasma concentration (ng/mL), mean ± SD | 275.3 ± 208.1 | 398.2 ± 240.3 | **0.001** | 242.3 ± 183.3 | 338.7 ± 156.4 | **0.019** |
| ^d^C/D ratio (ng/mL/mg/day), mean ± SD | 1.4 ± 1.1 | 1.2 ± 0.9 | **0.007** | 1.7 ± 1.4 | 1.5 ± 1.7 | 0.24 |
| ^e^Concomitant medications |  |  |  |  |  |  |
| Other antipsychotics, n (%) | 113 (52.1) | 442 (64.1) | **0.002** | 5 (20.0) | 12 (22.6) | 1.00 |
| Benzodiazepines, n (%) | 28 (12.9) | 159 (23.0) | **0.001** | 14 (56.0) | 29 (54.7) | 1.00 |
| Mood stabilizers, n (%) | 29 (13.4) | 136 (19.7) | 0.035 | 0 (0) | 6 (11.3) | 0.17 |
| Antidepressants, n (%) | 67 (30.9) | 130 (18.8) | **0.001** | 9 (36.0) | 20 (37.7) | 1.00 |
| Anticholinergics, n (%) | 15 (6.9) | 48 (7.0) | 0.98 | 3 (12.0) | 15 (28.3) | 0.15 |
| Laxatives, n (%) | 3 (1.4) | 29 (4.2) | 0.05 | 0 (0) | 14 (26.4) | **0.003** |
| ^f^CGI-S, mean ± SD | 5.1 ± 0.7 | 5.2 ± 0.8 | 0.24 | NA | NA | NA |
| ^f^Responders (GCI-I≥2), n (%) | 18 (12.1) | 64 (12.6) | 0.85 | NA | NA | NA |
| ^f^Responders (GCI-I≥3), n (%) | 51 (34.2) | 180 (35.6) | 0.76 | NA | NA | NA |
| ^g^Side effects, n (%) | 23 (18.9) | 90 (20.6) | 0.67 | NA | NA | NA |
| ^g^UKU side effects rating scale |  |  |  | NA | NA | NA |
| Psychic, n (%) | 8 (6.6) | 16 (3.7) | 0.16 | NA | NA | NA |
| Neurologic, n (%) | 9 (7.4) | 26 (5.9) | 0.57 | NA | NA | NA |
| Autonomic, n (%) | 7 (5.7) | 38 (8.7) | 0.29 | NA | NA | NA |
| Other, n (%) | 6 (4.9) | 9 (2.1) | 0.08 | NA | NA | NA |

Bold number indicates significant difference after Holm-Bonferroni multiple comparison correction.

AGATE: Arbeitsgemeinschaft Arzneimitteltherapie bei psychiatrischen Erkrankungen; CGI-S: Clinical Global Impression – Severity; C/D ratio – concentration to dose ratio; NA: not available; SD: standard deviation, UKU: Udvalg for kliniske undersogelser.

^a^Data was missing for 9 (1.5%) patients in AGATE Dataset

^b^Data was missing for 115 (18.8%) patients in AGATE Dataset and 9 (11%) in Lausanne dataset.

^с^Data was missing for 126 (20.6%) patients in AGATE Dataset and 7 (9%) in Lausanne Dataset.

^d^Data was missing for 14 (18%) patients in Lausanne Dataset.

^e^Data was missing for 13 (17%) patients in Lausanne Dataset.

^f^Data was missing for 222 (36.2%) patients in AGATE Dataset.

^g^Data was missing for 274 (44.7%) patients in AGATE Dataset.

# **Supplementary table S6:** Quality assessment of included cohorts with data on effectiveness or safety of clozapine treatment (poor, fair or good)

| **Study**  **(Author, year)** | **Quality of studies** | | | | |
| --- | --- | --- | --- | --- | --- |
|  | **Selection (0-5)** | **Comparability (0-2)** | **Outcome (0-2)** | **Overall Judgement** |  |
| Takeuchi (ZHH Cohort), 2016 | 4 | 1 | 1 | Good |  |
| de Filippis, 2021 | 3 | 1 | 1 | Good |  |
| Kitagawa (Yamanashi Cohort), 2021 | 4 | 2 | 1 | Good |  |
| Kitagawa (Okayama Cohort), 2021 | 4 | 1 | 1 | Good |  |
| Leclerc, 2021 | 3 | 1 | 0 | Poor |  |
| AGATE cohort, unpublished | 5 | 2 | 1 | Good |  |

AGATE: Arbeitsgemeinschaft Arzneimitteltherapie bei psychiatrischen Erkrankungen; ZHH: Zucker Hillside Hospital
