# Supplementary figures and images for: Clozapine once- versus multiple-daily dosing: a two-center cross-sectional study, systematic review and meta-analysis
Source: Eur Arch Psychiatry Clin Neurosci. 2022 Dec 29;273(7):1567–78. doi: 10.1007/s00406-022-01542-1 (PMC10465369; doi:10.1007/s00406-022-01542-1)

A

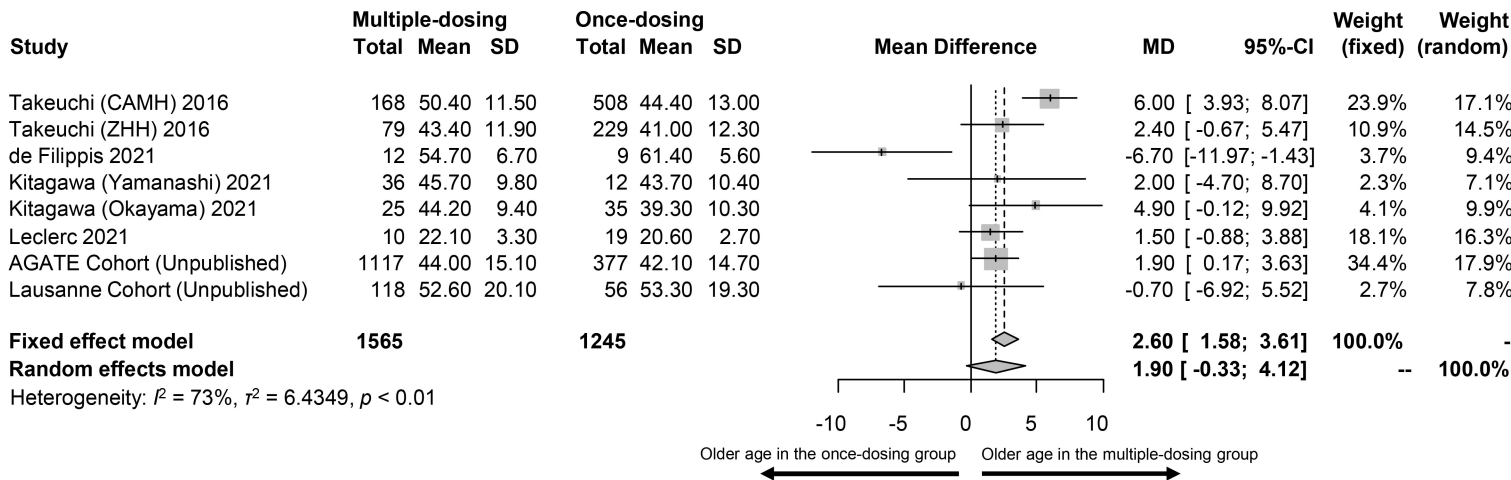

B

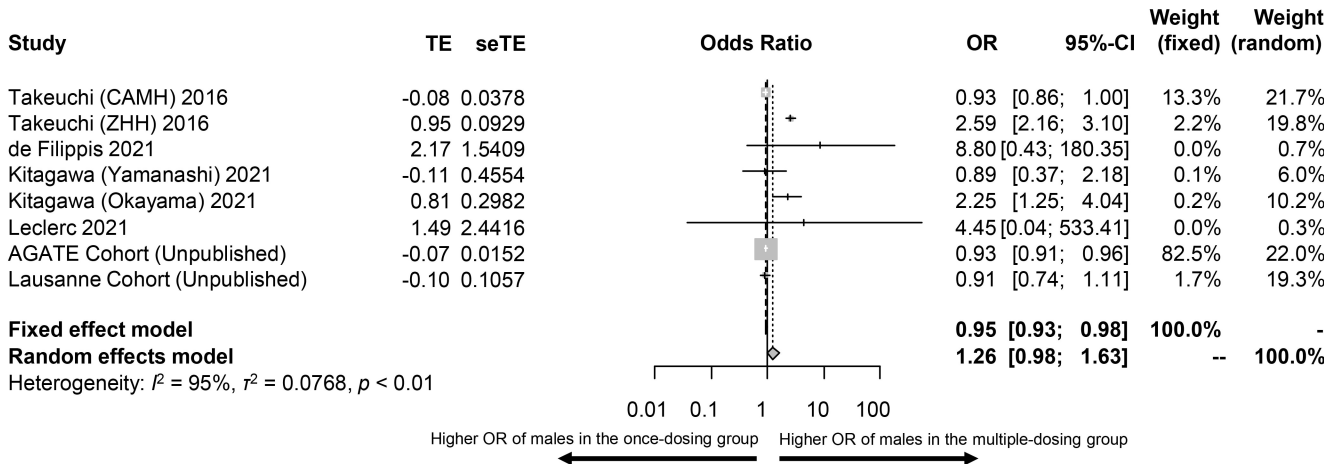

C

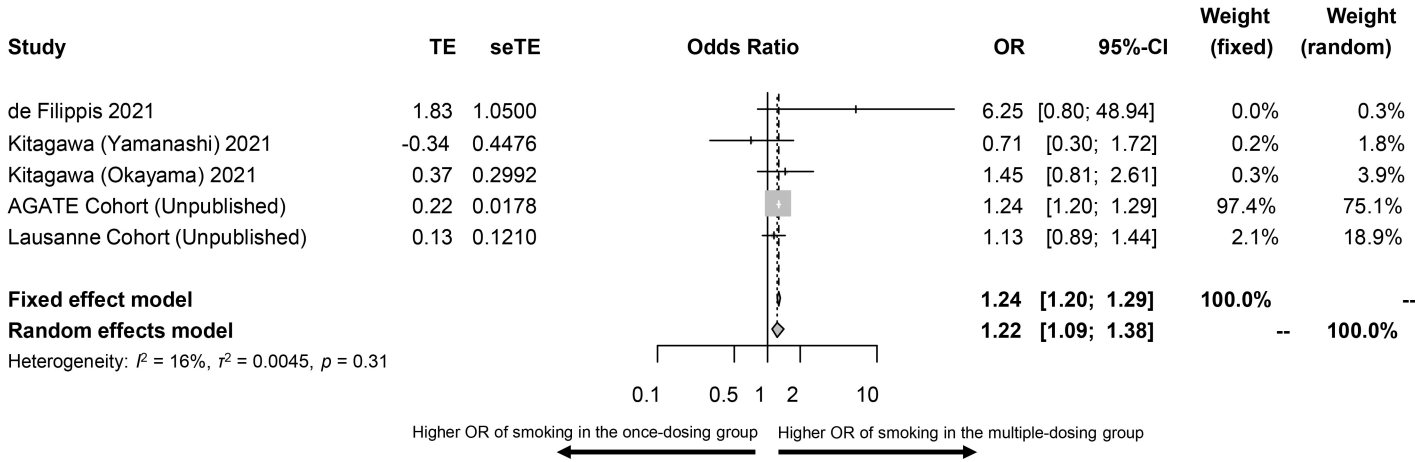

D

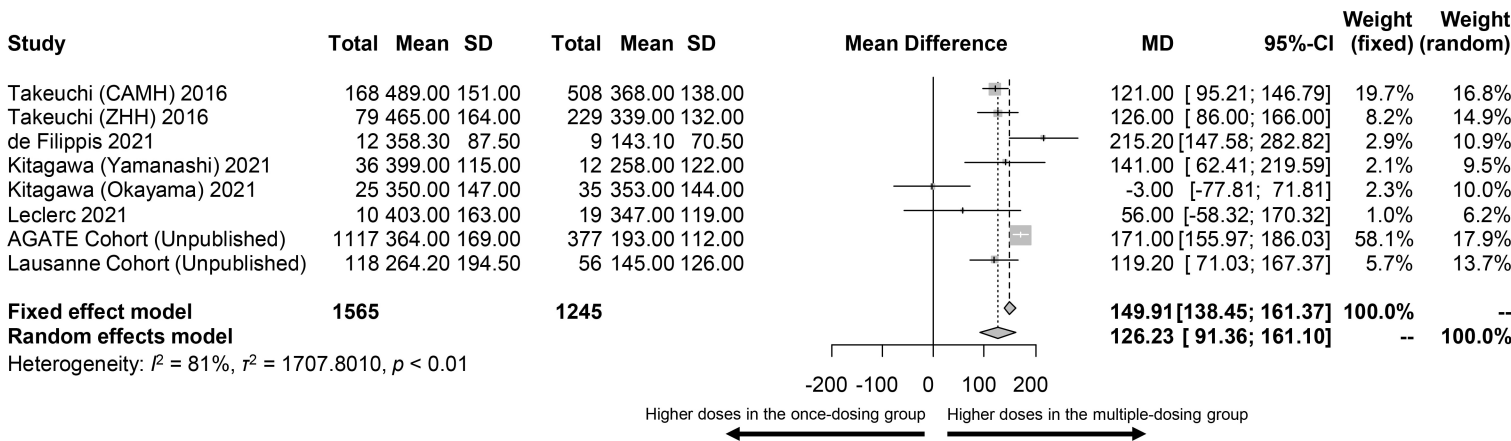

Supplement: Supplementary file 2 — Supplementary file2 (PDF 7344 KB) [file 406_2022_1542_MOESM2_ESM.pdf]

A

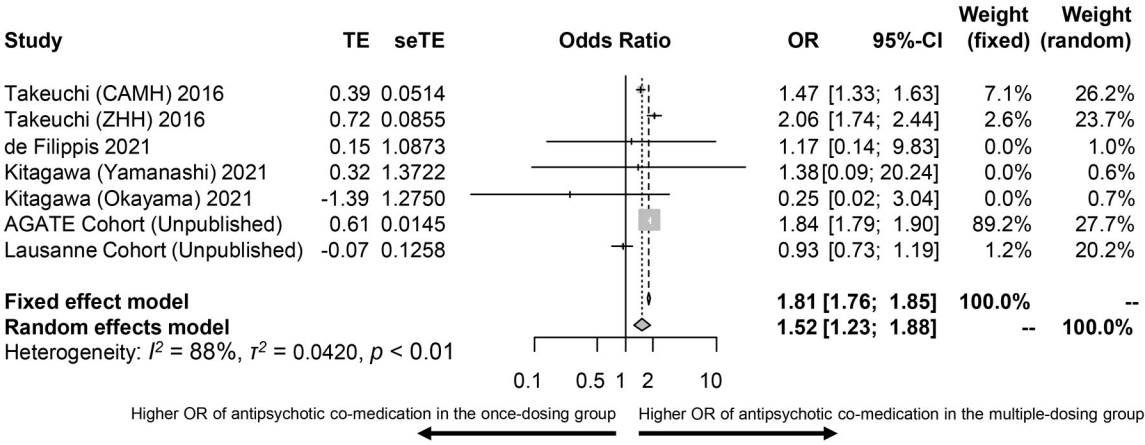

B

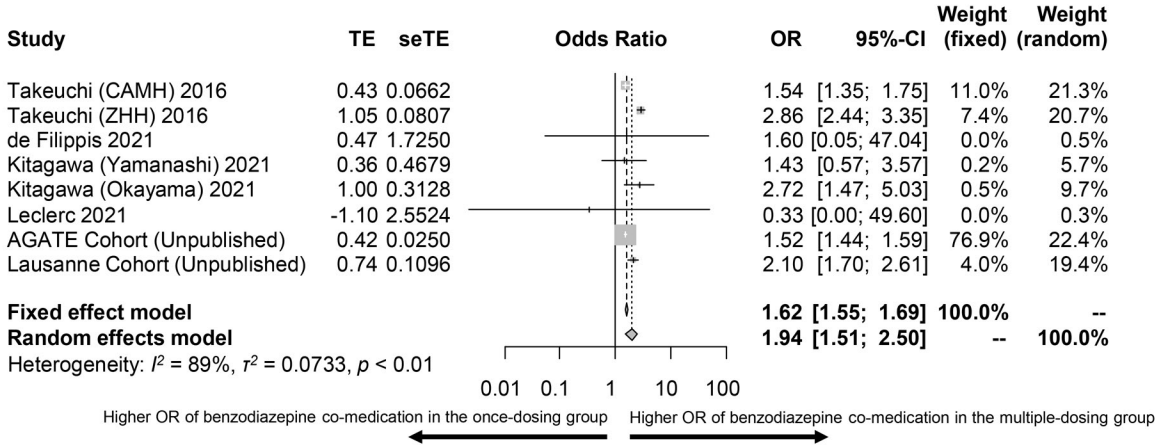

C

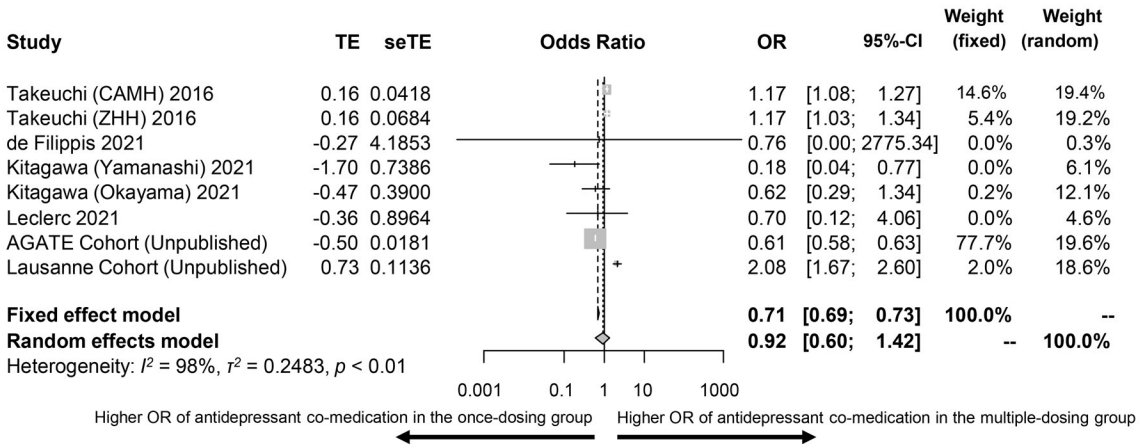

D

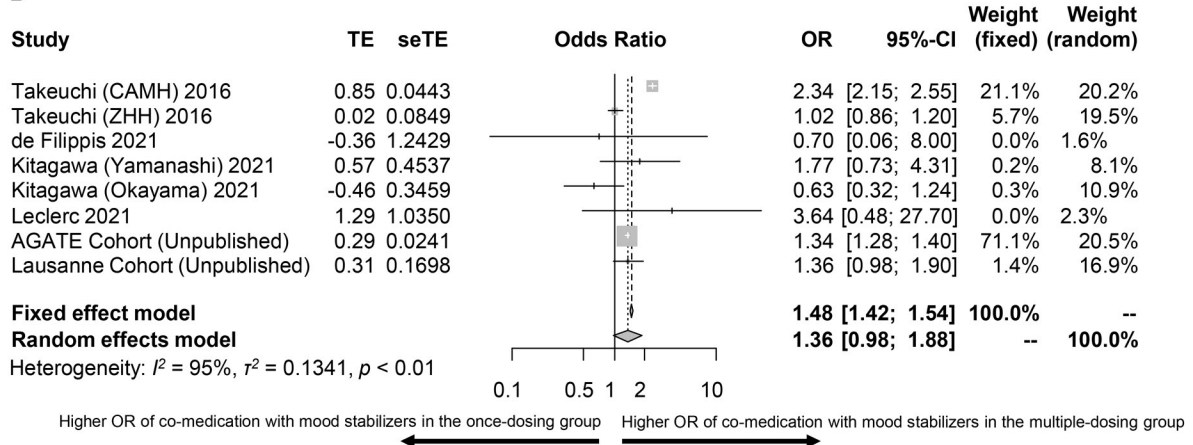

E

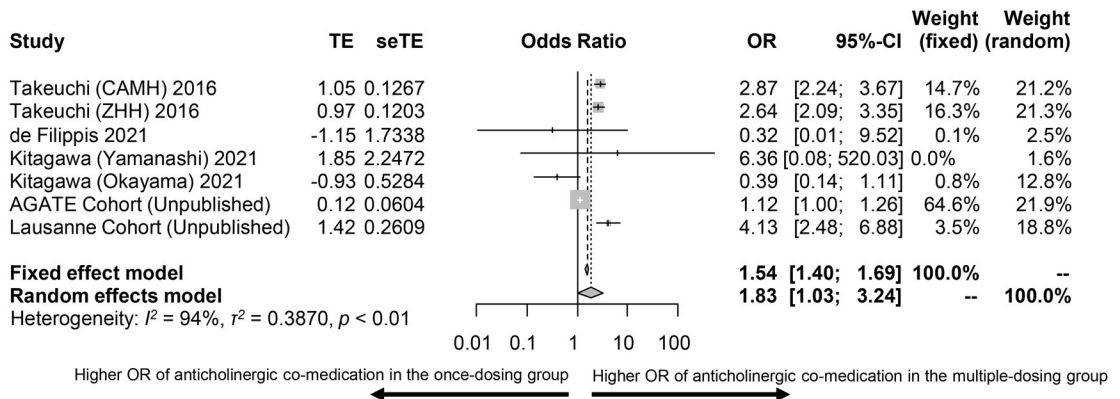

F

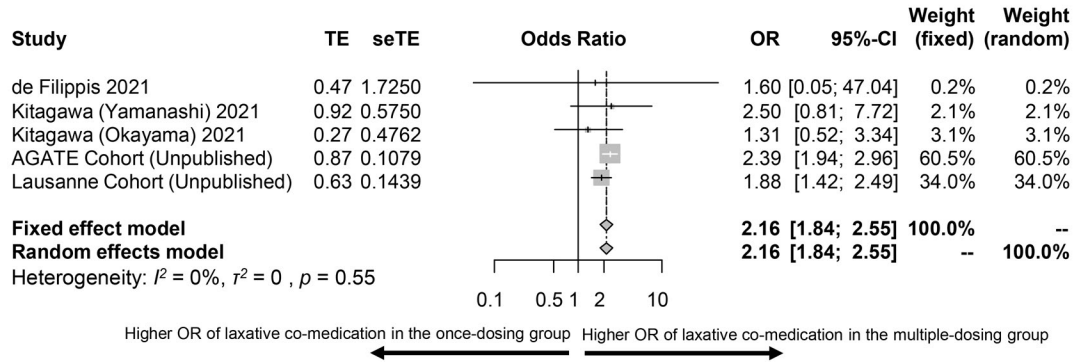

Supplement: Supplementary file 3 — Supplementary file3 (PDF 7609 KB) [file 406_2022_1542_MOESM3_ESM.pdf]
